# Household Hints and Recipes

**Published:** 1880-01

**Authors:** 


### Household Hints & Recipes

SEALING WAX FOR FRUIT CANS.—Melt together, yellow wax, one ounce; American vermillion, three ounces; gum shellac, five ounces; resin, sixteen ounces; run into moulds.

---

TO REMOVE INK FROM CARPETS, we would advise the application of a solution of citric and oxalic acids, followed by copious washing with water. This at least will not injure the wool; as to the colors of the carpet, that will depend entirely on the nature of the dye, but there is nothing else able to eradicate ink stains, that will have less effect on fast colors.

---

JAPANESE CEMENT.—Mix the best powdered rice with a little cold water, then gradually add boiling water until a proper consistence is acquired, being careful to keep it well stirred all the time; lastly, it must be boiled for one minute in a clean saucepan. This glue is beautifully white and almost transparent, for which reason it is well adapted for fancy paper work, which requires a strong and colorless cement.

---

TO PLATE IRON CASTINGS WITH COPPER WITHOUT A BATTERY, the usual way is to clean them thoroughly with dilute sulphuric or muriatic acid, and to immerse them for a short time in a solution of sulphate of copper, rendered acid with a small quantity of muriatic acid. The coat thus obtained is extremely thin and wears away in a short time, but such is the process employed for cheap household articles that are bronzed more with a view to sell them than to give them a permanent coat of copper.

---

TO PROTECT IRON FROM RUST.—Iron can be protected from rust and made very pleasing in color by a method invented by Mr. Dode. He coats the surface with a thin film of borate of lead, in which some oxide of copper has been dissolved, and some scales of precipitated platinum held in suspension, by means of a brush or a bath. He then heats the composition until it is diffused. The result is a thin, glassy coating, which will withstand the action of sewer gases, dilute acids or alkalies, and the heat of a kitchen fire. If all be true that is said of this "platinized iron," as it is called, it will find numerous applications.—*Kansas City Rev. of Sci. and Ind.*

BEST BLACKING FOR BOOTS AND SHOES.—Ivory black 1½ oz.; treacle, 1½ oz.; sperm oil, 3 drachms; strong oil of vitriol, 3 drachms; common vinegar, ½ pint. Mix the ivory black, treacle and vinegar, then the sperm oil and oil of vitriol separately, and add them to the other mixtures. This will be found an excellent and useful receipt.—*Leather Trades' Circular.*

---

CEMENT FOR UNITING METAL TO GLASS.—Dissolve one pound of shellac in a pint of strong methylated spirit (alcohol containing 10 per cent. of wood spirit), to which is added 0.05 part of solution of India-rubber in carbon disulphide.

Or, take two ounces of a thick solution of glue, and mix with one ounce of linseed oil varnish, or ½ ounce of Venice turpentine. Boil together and agitate.

The pieces cemented should be fastened for 50 to 60 hours to get fixed.—*Monthly Mag. of Pharm.*

---

TO REMOVE SPOTS FROM VARNISHED ARTICLES.

It depends altogether on the nature of the substance which caused the stain. In the absence of any specific knowledge of this point, we quote a method recommended in a German polytechnic journal. Make a mixture of equal parts of linseed oil, alcohol and turpentine, *slightly* moisten a rag with it, and rub the spots until they disappear. Then polish the spot with ordinary blotting paper. Varnish injured by heat can hardly be restored in any other way than by removing it and applying a fresh coat.

---

INFLUENCE OF GASLIGHT ON THE EYE.—The German Minister of Instruction, on a recent report on the influence of gaslight on the eye, concludes that no evil results follow a moderate use of gas, if the direct action of the yellow flame on the eye is prevented. Grave objections he makes to the use of zinc or lead shades, most evils affecting the eye being traceable to them. Their use, it is said, inevitably tends to blindness or inflammation, or other harmful effects. The milky white glass shade is the best, as it distributes the light and has a grateful effect on the eye. The burner should not be close to the head, as congestions of the forehead and headaches result from the radiated heat. The glass plate below the gas is especially useful for the purpose; as it causes an equal distribution of the light—necessary where a number are working at one burner—prevents the radiation of heat, and tends to a steady illumination by shielding the flame from currents of air. In cases of highly inflamed eyes, he recommends dark blue globes.

---

SOOT TEA FOR ROSES.—Get some soot from a chimney or stove where wood is used for fuel, put into an old pitcher and pour hot water upon it. When cool use it to water your plants every few days. When it is all used, fill up the pitcher again with hot water. The effect upon plants, especially upon roses that have almost hopelessly deteriorated, is wonderful in producing a rapid growth of thrifty shoots, with large, thick leaves and a large number of richly tinted roses. Never despair of a decayed rose-bush until this has been tried.—*Franklin County Times.*

---

TRANSPARENT PAPER.—A varnish made of Canadian balsam, dissolved in turpentine, supplies a most valuable means of making paper transparent. The mode by which this is most satisfactorily accomplished, is by applying a pretty thin coating of this varnish to the paper, so as to permeate it thoroughly, after which it is to be coated on both sides with a much thicker sample. The paper is kept warm by performing the operation before a hot fire, and a third or even fourth coating may be applied until the texture of the paper is seen to merge into a homogeneous translucency. Paper prepared according to this process is said to come nearer than any other to the highest standard of perfection in translucent paper. Care must be used in making, as the materials are highly inflamable.

---

LONG-MADE BEEF TEA.—Get two or three pounds of shin of beef; remove all the skin and the marrow from the bone; cut the meat into small pieces, and have the bone broken up. Take also a knuckle of veal—that is, just the knuckle bone; have it broken up, and put all into a strong earthen jar. Place the jar in a large saucepan of boiling water, and tie the cover down with a piece of stout brown paper, using neither salt nor pepper. Let it boil slowly all day. When done, the jar will be filled with meat gravy; strain this, and when cold it will be a strong jelly. In summer this may be served cold, and in winter pour hot water over a portion, and you have beef tea. This will keep a week in summer, in a cool place, and much longer in winter.

---

TO CLEAN OLD ENGRAVINGS.—There are many ways suggested for accomplishing this object, but the following plan, if carefully attended to, gives good results. Place the prints, one or two at a time, in a shallow dish, and pour water over them until they are completely soaked or saturated with it; then carefully pour off the water, and pour on them a solution of chloride of lime (one part liquor calcis chloratæ to 39 parts of water.) As a general rule, the stains disappear as if by magic, but occasionally they are obstinate. When that is the case, pour on the spots pure liquor calcis chloh ratœ, and if that does not succeed, add a little dilute nitro-muriatic acid. There is no print that will not succumb to this treatment; in fact, as a rule, they become too white. As soon as they are clean they must be carefully washed with successive portions of water until the whole of the chloride is got rid of. They should then be placed in a solution of isinglass or glue. Many collectors color this solution with coffee grounds, etc., to give a yellow tint to the engraving. They should then be dried between white blotting paper, either in a press or under a heavy book, and finally ironed with an ordinary flat iron to restore the gloss, placing clean paper between the iron and the print. Grease stains are much more difficult to remove. Benzine is the best for this purpose. Small grease spots may be removed by powdered French chalk being put over them, a piece of blotting paper over the chalk, and a hot iron over that. To clean manuscripts the chloride of lime will not do, as the writing will not stand its action as will the carbon printing ink. There is another kind of stain in the form of brown spots. which is often caused in framed engravings by the absorption of the coloring matter from knots in the wood behind. Only clear stuff should be used for backing, and to preclude the possibility of stains, thick paper or pasteboard should be placed between the engraving and the wood. To remove surface dirt from engravings and mezzotints the most effectual plan is to use common bookbinder's paste, which is to be applied with a paste brush both to the back and front of the print. The paste will take up the whole of the dirt, which will come away with the paste when it is removed with water. A bath of plain water completes the operation, from which the print will emerge as fresh as when first issued.

